# Supplementary material for: Metabolic contrast agents produced from transported solid 13C-glucose hyperpolarized via dynamic nuclear polarization
Source: Commun Chem. 2021 Jun 23;4:95. doi: 10.1038/s42004-021-00536-9 (PMC9814755; doi:10.1038/s42004-021-00536-9)
Supplement: Supplementary file 1 — Description of Additional Supplementary Files [file 42004_2021_536_MOESM1_ESM.pdf]

# Description of Additional Supplementary Files

**File Name:** Supplementary Movie 1

**Description**: The movie shows a demonstration of the extraction, transport and off-site dissolution of a long-relaxing hyperpolarized glucose sample
