# Supplementary material for: Metabolic contrast agents produced from transported solid 13C-glucose hyperpolarized via dynamic nuclear polarization
Source: Commun Chem. 2021 Jun 23;4:95. doi: 10.1038/s42004-021-00536-9 (PMC9814755; doi:10.1038/s42004-021-00536-9)
Supplement: Supplementary file 2 — Supplementary Information [file 42004_2021_536_MOESM2_ESM.pdf]

<u>**SUPPLEMENTARY MATERIAL**</u>

# Metabolic contrast agents produced from transported solid $^{13}$C-glucose hyperpolarized via Dynamic Nuclear Polarization

Andrea Capozzi [1,2*], Jan Kilund [2], Magnus Karlsson [2], Saket Patel [2], Arthur Cesar Pinon [2], François Vibert [3], Olivier Ouari [3], Mathilde H. Lerche [2], and Jan Henrik Ardenkjær-Larsen [2]

[1] *LIFMET, Department of Physics, EPFL, Station 6 (Batiment CH), 1015 Lausanne (Switzerland)..*

[2] *HYPERMAG, Department of Health Technology, Technical University of Denmark, Building 349, 2800 Kgs Lyngby (Denmark).*

[3]*Institut de Chimie Radicalire Aix-Marseille Université, CNRS, ICR UMR 7273, 13397 Marseille Cedex 20 (France).*

**Corresponding author**

*Dr. Andrea Capozzi

EPFL SB IPHYS LIFMET

CH F0 632 (Bâtiment CH)

Station 6

CH-1015 Lausanne

T: +41 21 693 05 88

Email: andrea.capozzi@epfl.ch

ORCID: 0000-0002-2306-9049

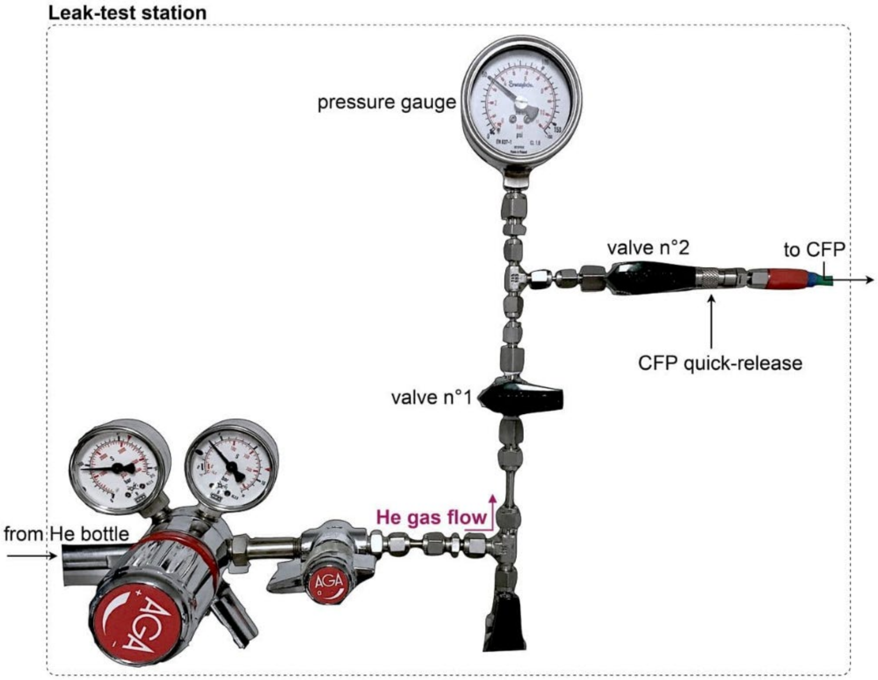


**Supplementary Figure 1. Leak-test station.** After loading the frozen pellets inside the sample vial, sealing and immersing it in liquid nitrogen, the quick-release is connected on *valve 2* and He gas gently blown to flush the CFP inner volume. Then, the one-way valve is closed with a plug and the pressure inside the CFP increased to 4 bar. *valve 1* is then closed and the pressure monitored via the *pressure gauge* for 5 min. If no pressure drop is observed the leak-test is considered successful. The plug is finally removed from the one-way valve and the CFP disconnected from the leak-test station to be loaded inside the polarizer.

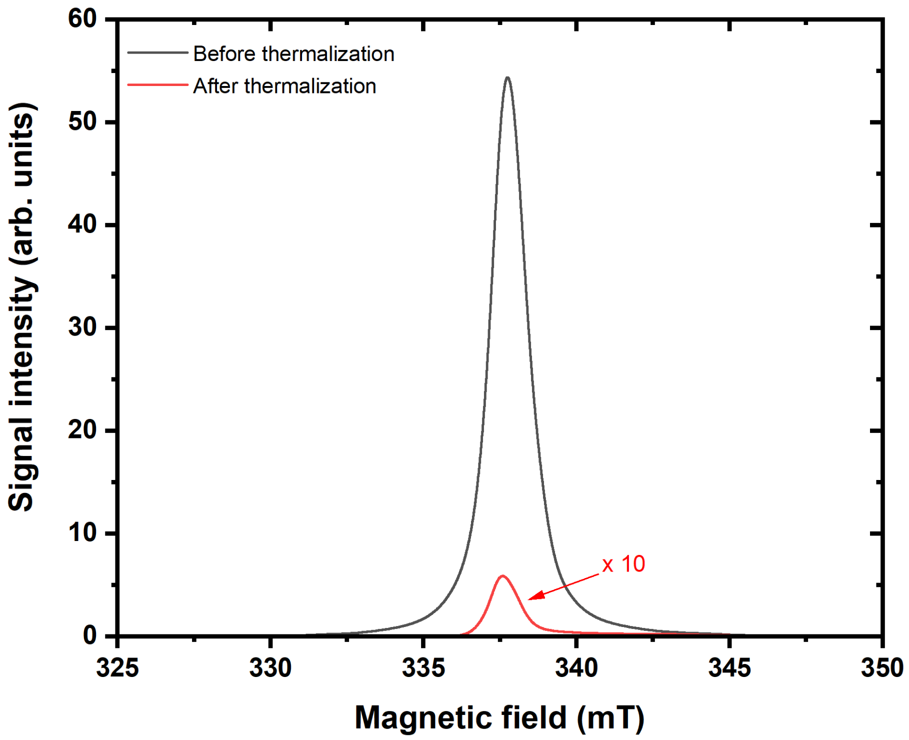


**Supplementary Figure 2. Radical leftover after quenching procedure.** X-band ESR spectrum measured at 77 K for a sample before (black line) and after (red line) the thermalization/radical quenching procedure. The radical leftover calculates approximately 1% of the initial value.

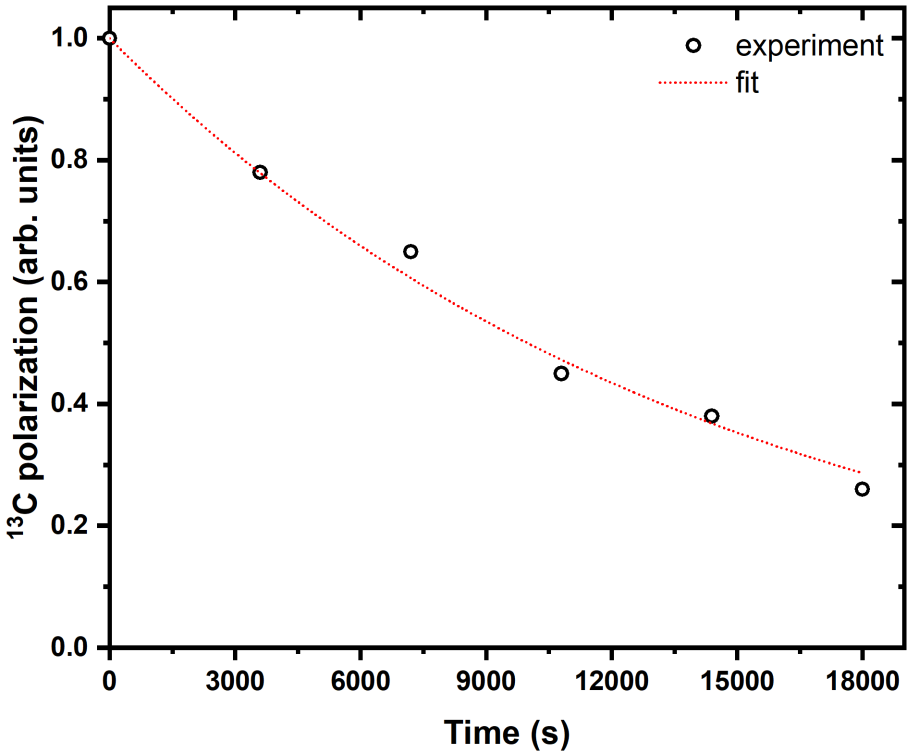


**Supplementary Figure 3. Sample relaxation after thermalization at 4.2 K and 1 T.** The graph reports the results of the manual field cycling implemented inside the dDNP polarizer to measure the $^{13}C$ glucose $T_1$ at 1 T and 4.2 K. Each black circle corresponds to the NMR spectrum double-integral measured at 6.7 T and 4.2 K after having left the sample relaxing 25 cm above the NMR coil. The red dotted curve represents a mono-exponential fit (time constant = 4.0±0.5 h, $R^2$ = 0.97)

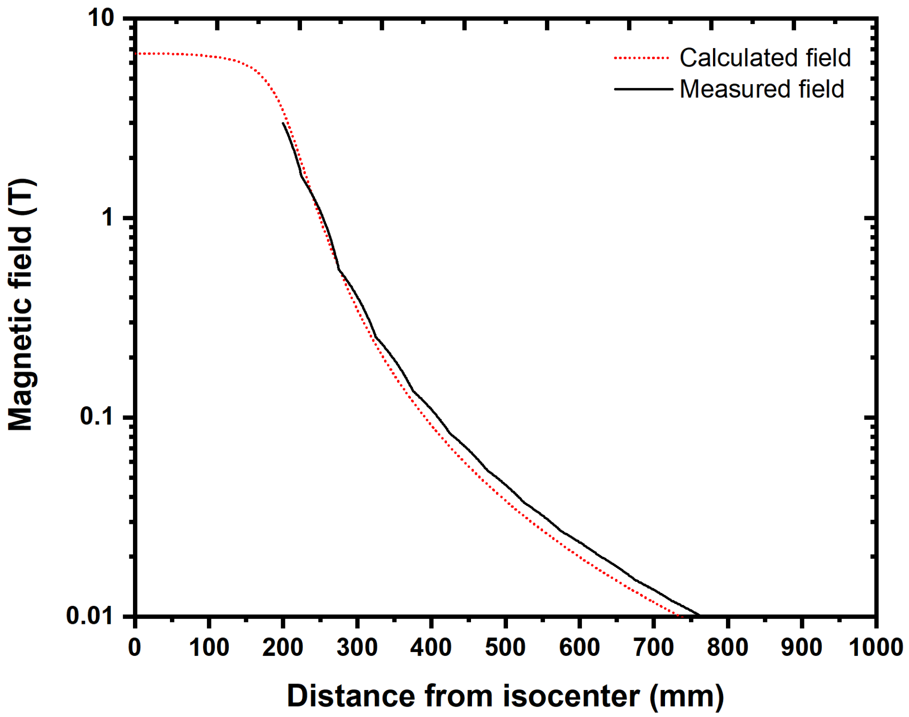


**Supplementary Figure 4. Measurements and simulation of the polarizer stray magnetic field.** The dDNP polarizer stray field was measured (black line), in steps of 10 cm, from the loading chamber to 20 cm above the isocenter. The latter corresponded to the Hall probe saturation value (i.e. 3 T). The stray field, as function of the distance from the isocenter *z*, was also calculated (red dotted line) using the formula for a real solenoid $B(z) = \frac{B_0}{2}\left(\frac{\frac{l}{2}-z}{\sqrt{R^2+\left(\frac{l}{2}-z\right)^2}} + \frac{\frac{l}{2}+z}{\sqrt{R^2+\left(\frac{l}{2}-z\right)^2}}\right)$ with length $l = 400\ mm$ and radius $R = 50\ mm$. The agreement between measurements and calculation was as good as $R^2 = 0.98$.

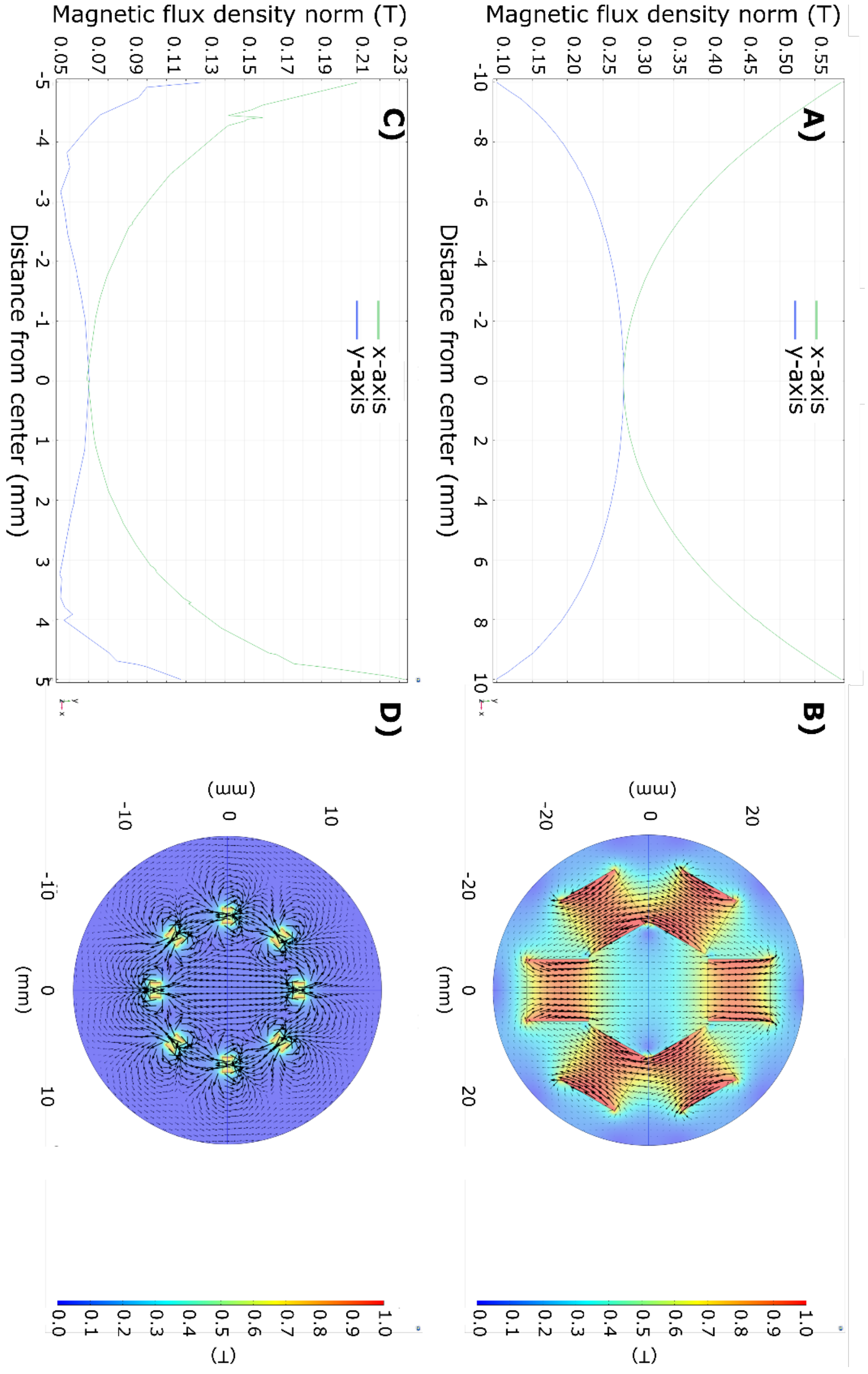


**Supplementary Figure 5. Finite elements simulations of the different Halbach arrays placed inside the magnetic enforced DNP probe. Part 1.** The Ampere's law, in case of no running currents, was solved in COMSOL 5.4 considering permanent magnets with residual magnetization

of 1.4 T (according to manufacturer specifications); the surrounding material was air (i.e. $\mu_r = \varepsilon_r = 1$). Panel (A) and (B) represent the graph and magnetic flux density map for the hexagonal Halbach array placed around the loading chamber, respectively. Panel (C) and (D) represent the graph and magnetic flux density map for the small octagonal Halbach arrays placed inside the KF16 half nipples, respectively.

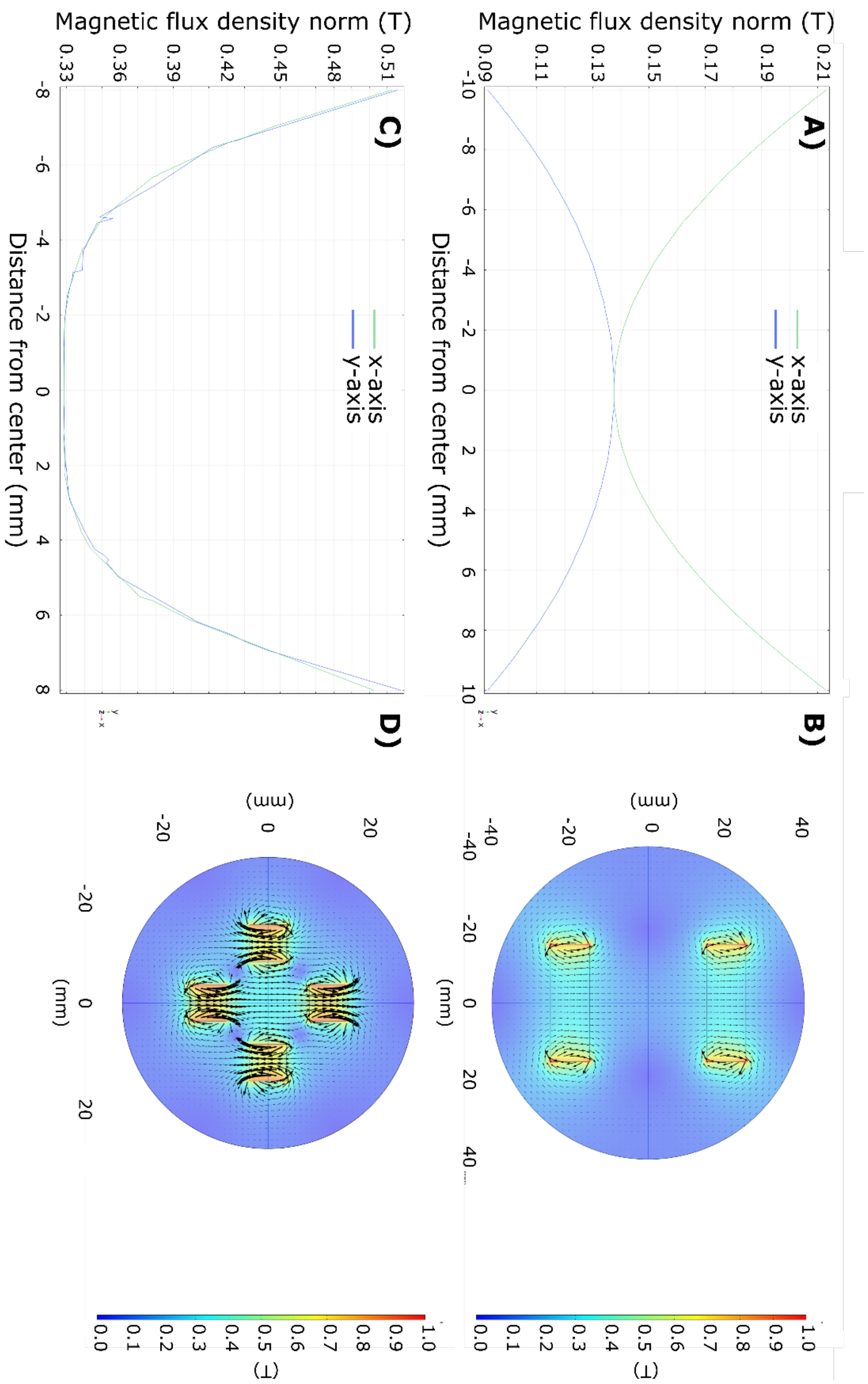


**Supplementary Figure 6. Finite elements simulations of Halbach arrays placed inside the magnetic enforced DNP probe. Part2.** The Ampere's law, in case of no running currents, was

solved in COMSOL 5.4 considering permanent magnets with residual magnetization of 1.4 T (according to manufacturer specifications); the surrounding material was air (i.e. $\mu_r = \varepsilon_r = 1$). Panel (A) and (B) represent the graph and magnetic flux density map for the 2 elements Halbach array around the gate valve, respectively. Panel (C) and (D) represent the graph and magnetic flux density map for the four elements Halbach array placed around the probe stem, respectively.

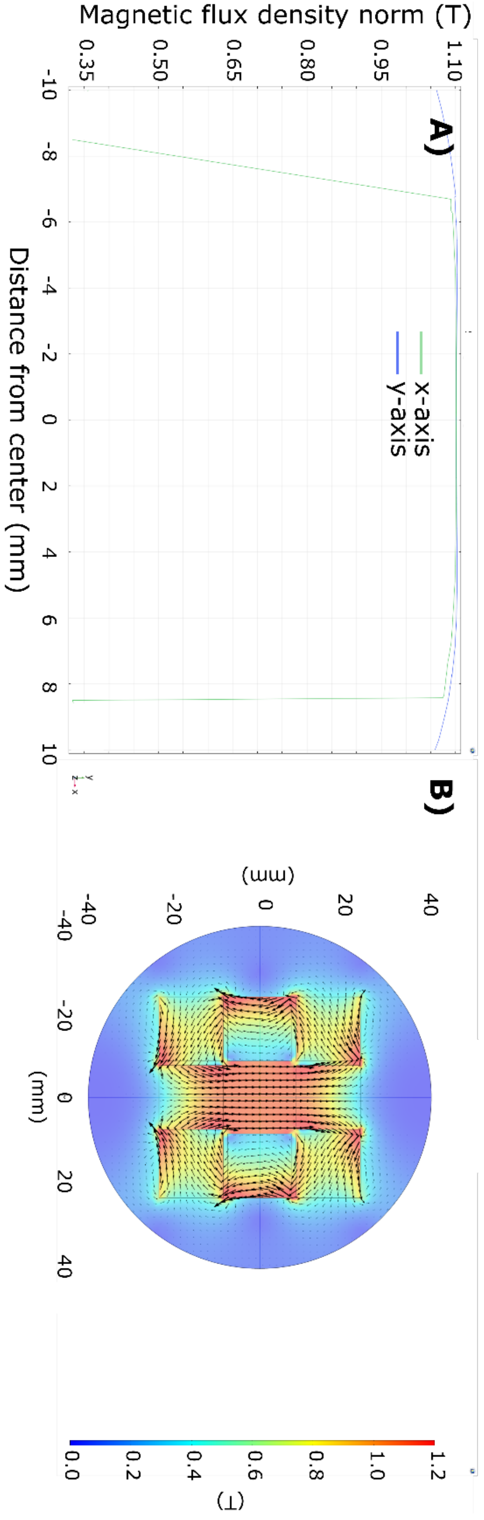


**Supplementary Figure 7. Finite element simulation for storage magnet.** The Ampere's law, in case of no running currents, was solved in COMSOL 5.4 considering permanent magnets with

residual magnetization of 1.4 T (according to manufacturer specifications); the surrounding material was air (i.e. $\mu_r = \varepsilon_r = 1$). Panel (A) and (B) represent the graph and magnetic flux density map for the 9 elements Halbach array forming the storage magnet.

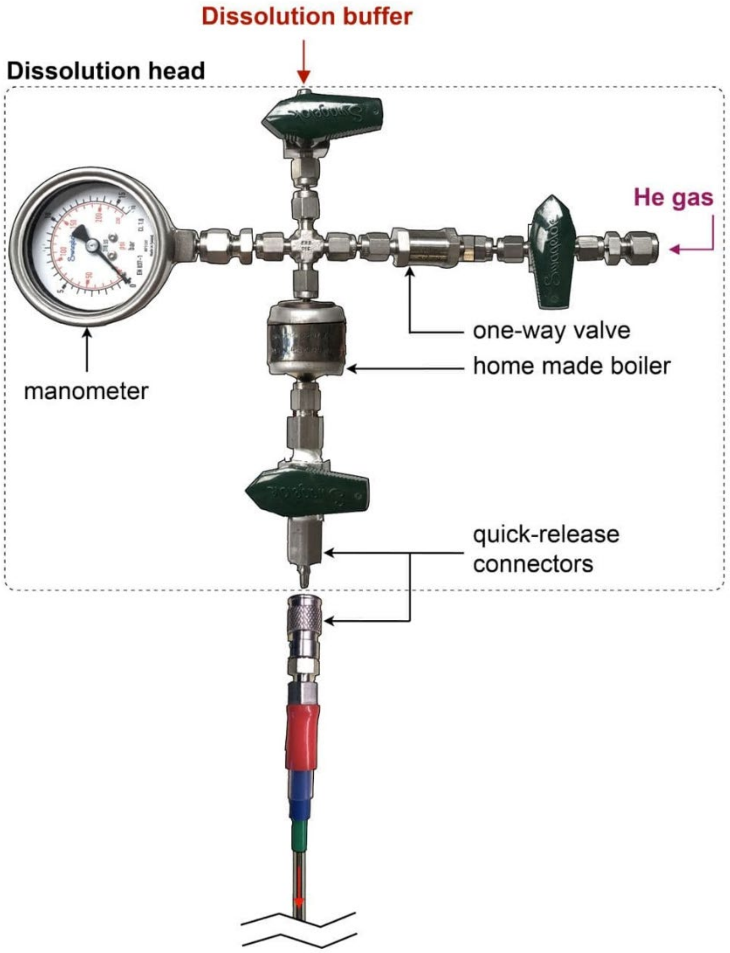


**Supplementary Figure 8. Compact dissolution station head.** Illustration of the compact dissolution station head used to obtain the HP solution after transport. The dissolution buffer is loaded inside the boiler and pressurized to 4 bar using He gas. The buffer is heated up to 180 °C and kept at this temperature using a PDI controller. Once on site with the transport device, the CFP quick connect is plugged to the boiler and the hot buffer released.
